# Food-Derived Carbon Dots: Formation, Detection, and Impact on Gut Microbiota

**DOI:** 10.3390/foods14172980

**Published:** 2025-08-26

**Authors:** Duyen H. H. Nguyen, Hassan El-Ramady, Gréta Törős, Arjun Muthu, Tamer Elsakhawy, Neama Abdalla, Walaa Alibrahem, Nihad Kharrat Helu, József Prokisch

**Affiliations:** 1 Institute of Animal Science, Faculty of Agricultural and Food Sciences and Environmental Management, Biotechnology and Nature Conservation, University of Debrecen, 138 Böszörményi Street, 4032 Debrecen, Hungary; nguyen.huu.huong.duyen@agr.unideb.hu (D.H.H.N.); toros.greta@agr.unideb.hu (G.T.); 2 Institute of Life Sciences, Vietnam Academy of Science and Technology, 9/621 Vo Nguyen Giap Street, Linh Trung Ward, Thu Duc City 700000, Ho Chi Minh, Vietnam; 3 Doctoral School of Nutrition and Food Science, University of Debrecen, 4032 Debrecen, Hungary; arjun.muthu@agr.unideb.hu; 4 Soil and Water Department, Faculty of Agriculture, Kafrelsheikh University, Kafr El-Sheikh 33516, Egypt; 5 Doctoral School of Animal Science, University of Debrecen, 4032 Debrecen, Hungary; 6 Institute of Agricultural Chemistry and Soil Science, Faculty of Agricultural and Food Sciences and Environmental Management, University of Debrecen, 138 Böszörményi Street, 4032 Debrecen, Hungary; 7 Agricultural Microbiology Research Department, Soils, Water and Environment Research Institute (SWERI), Agriculture Research Center (ARC), Giza 12112, Egypt; drelsakhawyg@gmail.com; 8 Plant Biotechnology Department, Biotechnology Research Institute, National Research Centre, 33 El Buhouth St., Dokki, Giza 12622, Egypt; neama_ncr@yahoo.com; 9 Doctoral School of Health Sciences, University of Debrecen, Egyetem Tér 1, 4028 Debrecen, Hungary; walaaeb@mailbox.unideb.hu (W.A.); nihad.kharrat.helu@mailbox.unideb.hu (N.K.H.)

**Keywords:** carbon nanodots, food processing, microbiome–nanomaterial interaction, intestinal homeostasis

## Abstract

Food-derived carbon dots (F-CDs) are a novel class of carbon-based nanomaterials unintentionally generated during common thermal food processing techniques, such as baking, roasting, frying, and caramelization. These nanostructures exhibit unique optical and chemical properties, including photoluminescence, high aqueous solubility, and tunable surface functionality, making them increasingly relevant to both food science and biomedical research. Recent studies have highlighted their ability to interact with biological systems, particularly the gut microbiota, a critical determinant of host metabolism, immunity, and overall health. This review critically summarizes the current understanding of F-CDs, including their mechanisms of formation, analytical detection methods, and physicochemical properties. It explores their biological fate in the gastrointestinal tract, encompassing absorption, distribution, metabolism, and excretion, with a focus on their stability and cellular uptake. Special attention is given to the interaction between F-CDs and the gut microbiota, where evidence suggests both beneficial (e.g., anti-inflammatory, antioxidant) and detrimental (e.g., dysbiosis, inflammatory signaling) effects, depending on the CD type, dose, and exposure context. Additionally, this review addresses toxicological concerns, highlighting gaps in long-term safety data, standardized detection methods, and regulatory oversight. The dual role of F-CDs—as potential modulators of the microbiota and as emerging dietary nanomaterials with uncharted risks—underscores the need for further interdisciplinary research. Future efforts should aim to refine detection protocols, assess chronic exposure outcomes, and clarify structure–function relationships to enable the safe and responsible application of these nanomaterials in food and health contexts.

## 1. Introduction

Carbon dots (CDs) are emerging carbon-based nanomaterials recognized for their strong photoluminescence, solubility in water, chemical stability, and low toxicity to cells. These properties make them promising candidates for many applications, particularly those relating to bioimaging, sensing, environment monitoring, and nanomedicine [1].

Food-derived CDs can be broadly classified according to the type of dietary precursor and processing pathway involved in their formation. Carbohydrate-rich foods (e.g., cereals, baked goods) typically generate CDs through Maillard reaction intermediates and caramelization [2,3]. Protein-based foods (e.g., meats, dairy products) yield CDs via amino acid–sugar interactions (Maillard reaction), producing nitrogen-doped structures with distinct surface functionalities [4,5]. Lipid-based foods subjected to frying or roasting contribute CDs through lipid oxidation and thermal polymerization of fatty acids [6]. Acidic matrices, such as citrus juices or citric-acid-rich products, can also act as efficient precursors, where hydrothermal or microwave treatments produce oxygen-rich, highly fluorescent CDs [7]. Fermentation may further amplify CD formation by generating reactive intermediates that transform during heating [8]. This classification highlights the structural and chemical diversity of food-derived CDs and underscores how the precursor type and processing conditions shape their physicochemical behavior.

Among the various pathways, the Maillard reaction plays a central role in CD formation during food processing. Initiated by the interaction of reducing sugars and amino acids, the Maillard reaction produces early intermediates such as Amadori products, which, over time, evolve into highly conjugated aromatic compounds that cluster into nanoscale carbon-rich cores, forming CDs. This process is particularly prominent in protein- and carbohydrate-rich foods subjected to prolonged or high-intensity heating [9].

Besides Maillard pathways, other mechanisms contribute to CD formation. Lipid oxidation and polymerization generate CDs from fatty food components [10], while citric acid dehydration and carbonization in acidic matrices yield highly fluorescent, oxygen-rich nanoparticles under hydrothermal or microwave conditions [11,12]. Notably, fermentation can further enhance CD formation by generating reactive intermediates during subsequent heating [1]. As a result, food-derived CDs have been detected in a range of common dietary items, such as bread crusts [2], roasted coffee [13], grilled meats [4], and spices [14].

Although the presence of CDs in thermally processed foods is well established, current evidence does not conclusively demonstrate their toxicity to human health. In our previous work, we concluded that while CDs exhibit unique physicochemical properties, there is insufficient evidence to classify them as harmful under typical dietary exposure conditions [15]. However, the unintentional formation of CDs during food processing necessitates a deeper understanding of their interactions within the human body, particularly with the gut microbiota.

The gut microbiota, now recognized as a central determinant of overall health through its regulation of metabolism, immunity, and neurological function, represents a critical area of investigation [16]. Even if limited direct evidence exists, preliminary research suggests that CDs from food sources have the capability of influencing the gut microbiota through the alteration of microbial diversity, generation of reactive oxygen species, or an influence on the microbial metabolome through their redox properties and their functional groups on the surface [17]. For example, in vitro experimentation with biomass-based CDs has shown selective antibacterial activities, likely disrupting commensal–pathogen homeostasis and the production of short-chain fatty acids [10]. Similarly, polyphenols from natural compounds like ginseng and olivetol have also exhibited the potential to reorganize the gut microbiota in favor of probiotic superiority while suppressing pathobionts like *Prevotella*, indicating that dietary agents, if thermally modified, can also embody an analogous microbial interface profile [18,19].

Although research on food-derived CDs is still in its early stages, their routine presence in common foods and their physicochemical resemblance to known microbial modulators make them important candidates for investigation. This review critically addresses their formation, detection, biological fate, and microbiota-mediated health implications, while highlighting methodological challenges and gaps in risk assessment and regulation.

## 2. Formation of Food-Derived Carbon Dots (F-CDs)

**Figure 1:**
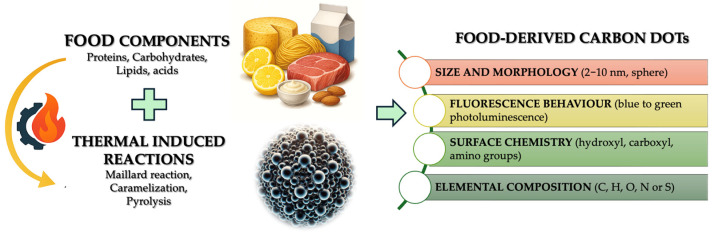
Physicochemical properties of carbon dots derived from food.

Food-derived carbon dots (F-CDs) are nanoscale carbon-based particles (1–10 nm) known for their fluorescence, high water solubility, and reactive surface chemistry. They have a potential range of applications in food science, materials engineering, and medicine [20]. Figure 1 illustrates how structural, elemental, and functional parameters vary across F-CDs. F-CDs can be synthesized from renewable natural extracts into functional biomaterials, aligning with the principles of green chemistry [21,22]. The green synthesis of CDs using food by-products, such as banana peels, and phytochemicals derived from them is a promising, eco-friendly method [9,23].

The formation of F-CDs generally occurs during the thermal processing of food, such as baking, roasting, frying, or pyrolysis. Several chemical pathways contribute to their synthesis:

- **Maillard Reaction:** The reaction between reducing sugars and amino acids generates Amadori products, which undergo further condensation and aromatization to form carbon-rich nanostructures. This process is especially significant under alkaline conditions and prolonged heating [24,25,26].
- **Caramelization:** The thermal degradation of sugars in the absence of amino acids can also yield CDs with characteristic surface functional groups [24].
- **Pyrolysis:** The low-oxygen thermal decomposition of organic matter produces graphitic and amorphous carbon domains, leading to CDs [27].
- **Lipid Oxidation and Polymerization:** Fatty acids and lipids undergo thermo-oxidative breakdown to form reactive carbon fragments, resulting in hydrophobic CDs with a lower emission efficiency [28].
- **Acidic Food Matrices:** Citric acid, abundant in citrus-based foods, readily forms CDs via dehydration, condensation, and carbonization under hydrothermal or microwave-assisted conditions [29].

Environmental conditions have a significant influence on the outcomes in this context. For example, acidity can result in oxygen-rich carbon dots that exhibit reduced luminescence, while moisture can impact heat distribution and thereby limit carbonization efficiency [30]. Additionally, salts and minerals may function as either catalysts or inhibitors, depending on their specific chemical properties of CDs [31]. Moreover, the oxidation state of the precursor and the chosen activation strategy play a crucial role in determining the distribution of surface functional groups, which, in turn, directly influence the optical and chemical properties of the CDs [32].

Some researchers were intensely focused on reaction mechanisms [32,33]. For instance, Hill and Galan (2017) examined how caramelization and lipid oxidation shape CD surface features [32], while Paloncýová et al. (2018) used molecular dynamics to study solvent effects, revealing that interlayer hydrogen bonding can compensate for reduced solvent interaction [33]; these facts can help us to understand the factors (chemical composition, solvent, environment) affecting production.

When it comes to precursors, biomolecules from food are essential. Carbohydrates offer structural variation and serve as sources of carbon [34,35], while proteins and amino acids contribute nitrogen and sulfur elements that improve both fluorescence and particle stability [36]. Lipid-based environments affect CD surface chemistry through oxidation, often resulting in hydrophobic and carbon-rich dots with diminished emission efficiency [28]. These distinct contributions highlight the importance of selecting appropriate starting materials.

The surface of these nanoparticles typically features functional groups, such as hydroxyl, carboxyl, and amino moieties, which improve solubility and biological activity [37]. Their elemental makeup consists mainly of carbon, hydrogen, and oxygen, with trace elements such as nitrogen, sulfur, phosphorus, and metals incorporated, depending on the precursor [38].

Recent studies by our group have demonstrated the presence of F-CDs in various foods—including coffee brews [13], oyster mushroom powder [38], pretzels [3], black pepper, turmeric, cysteine, clove, ginger, and chili spices [14]—using size-exclusion high-performance liquid chromatography with a fluorescence detector (HPLC-SEC-FD), a spectrofluorimeter, Raman spectroscopy, and Fourier Transform Infrared Spectroscopy (FTIR). The food safety of CDs was assessed in our published work [15]. Our findings also highlight the potential of valorizing food by-products to create CNDs, thereby contributing to a balanced gut microbiota and improved overall health outcomes [23]. Clear CDs should be produced from different food sources, and further investigations should be conducted to demonstrate their effectiveness in animal and human models, with an emphasis on the toxicological aspects of the final products.

Different synthesis routes are available, including bottom-up approaches (assembling structures from ions) and top-down techniques (breaking down bulk materials into nanoscale particles) [39]. A Parvin et al. (2024) demonstrated that the precursor type (e.g., citric acid, glucose, amino acids) and processing conditions such as the hydrothermal treatment temperature (150–250 °C) and reaction time (2–12 h) strongly influenced the particle size (ranging from 2 to 10 nm) and surface chemistry of the resulting quasi-spherical nanoparticles [40]. For instance, increasing the hydrothermal temperature from 180 °C to 220 °C led to a shift in emission maxima from ~430 nm to ~510 nm, accompanied by enhanced graphitization and nitrogen incorporation. The way CDs behave optically, such as exhibiting blue-to-green fluorescence under UV light, depends on factors including their internal structure, graphitic content, the presence of dopants, and the treatment of their surfaces [41]. Interestingly, higher thermal treatment temperatures have also been correlated with improved antioxidant capacities; for example, DPPH-radical-scavenging efficiency increased from ~40% at 180 °C to >70% at 220 °C, although such changes can also increase cytotoxicity in certain cell lines, reducing viability from ~95% to ~70% at doses above 200 µg/mL [42].

## 3. Detection and Characterization of Food-Derived CDs

The detection and characterization of food-derived carbon dots (CDs) are essential for understanding their formation mechanisms, physicochemical properties, and potential biological activities. Given the complex nature of food matrices, a multi-technique analytical approach is typically required to confirm the presence of CDs and differentiate them from other nanoscale or carbonaceous materials formed during thermal processing, as listed in Table 1, including the following:

- **Fluorescence spectroscopy** is among the most widely used methods for initial detection due to the inherent photoluminescent properties of CDs, which arise from their surface states, graphitic cores, or molecular fluorophores [43]. The excitation-dependent or independent emission spectra of CDs can be exploited to detect and semi-quantify their presence in processed food products. Complementary structural analysis is often performed using transmission electron microscopy (TEM), which enables the direct observation of particle size, morphology, and lattice fringes, typically revealing spherical particles in the 1–10 nm range [44].
- **Fourier-transform infrared spectroscopy (FTIR) and X-ray photoelectron spectroscopy (XPS)** are employed to identify surface functional groups such as hydroxyl, carboxyl, and amine moieties, which are critical for solubility, biological interactions, and fluorescence behavior [45,46].
- **Raman spectroscopy** further provides insights into the degree of graphitization by examining the D and G bands, indicative of disorder and sp^2^ hybridized carbon structures, respectively [47].
- **X-ray diffraction (XRD)** is used to assess crystallinity [48], while **dynamic light scattering (DLS)** offers hydrodynamic size distribution in aqueous environments [49].
- **High-performance liquid chromatography coupled with size-exclusion chromatography and fluorescence detection (HPLC-SEC-FD)** has proven particularly effective for identifying and quantifying CDs within complex food systems [14,23]. This method allows separation based on molecular size while simultaneously detecting fluorescent fractions.

Despite these advancements, a significant challenge remains in distinguishing CDs from other thermally induced fluorophores or nano-aggregates naturally present in food. The lack of standardized analytical protocols and certified reference materials hinders cross-study comparisons and reproducibility. Future research should focus on the development of harmonized detection workflows that integrate orthogonal methods, along with guidelines for minimum characterization standards, to enable more robust identification and evaluation of F-CDs.

**Table 1:** Standard techniques to analyze food-derived carbon dots (F-CDs).

Method	Principle	Advantages	Limitations	Application in Foods	Refs.
**Fluorescence Spectroscopy**	Measures light emission after excitation	Sensitive, non-destructive, real-time monitoring	May detect non-CD fluorophores; low specificity	General detection of CDs in processed foods	[49]
**TEM (Transmission Electron Microscopy)**	Visualizes particle size and morphology	High-resolution imaging, nanometer-scale detail	Sample prep intensive; non-representative bulk analysis	Size/morphology analysis in roasted/baked products	[44]
**FTIR** **(Fourier-transform Infrared Spectroscopy)**	Detects functional groups based on bond vibrations	Identifies surface chemistry; fast and accessible	Limited structural resolution; overlaps in complex matrices	Characterization of CDs from fruit peels, coffee	[46]
**XPS** **(X-ray Photoelectron Spectroscopy)**	Analyzes elemental composition and bonding states	Detailed surface elemental analysis	Surface-sensitive, expensive	Elemental profiling of CDs from spices	[45]
**Raman Spectroscopy**	Measures inelastic light scattering (D and G bands)	Probes graphitic structure and carbon disorder	Fluorescence interference; weak signal in some samples	Identifying graphitic features of CDs	[47]
**XRD** **(X-ray Diffraction)**	Determines crystalline phases and lattice structures	Reveals crystallinity; complementary to TEM	Not suitable for amorphous or low-crystalline materials	Assessing structure in food waste-derived CDs	[48]
**DLS** **(Dynamic Light Scattering)**	Measures hydrodynamic particle size in suspension	Simple, quick sizing in liquids	Sensitive to aggregates; poor resolution of small particles	Size analysis of aqueous CDs suspensions	[47]
**HPLC-SEC-FD (High Performance Liquid Chromatography–Size Exclusion Chromatography–Fluorescence Detection)**	Separates by size, detects fluorescence	High sensitivity, specific to fluorescent fractions	Requires standards and instrument calibration	Isolation of CDs from coffee, mushrooms, beer	[14,23]

## 4. Biological Fate of Food-Derived CDs

### 4.1. Absorption, Distribution, Metabolism, and Excretion (ADME) Profiling of Food-Derived Carbon Dots (F-CDs)

**Figure 2:**
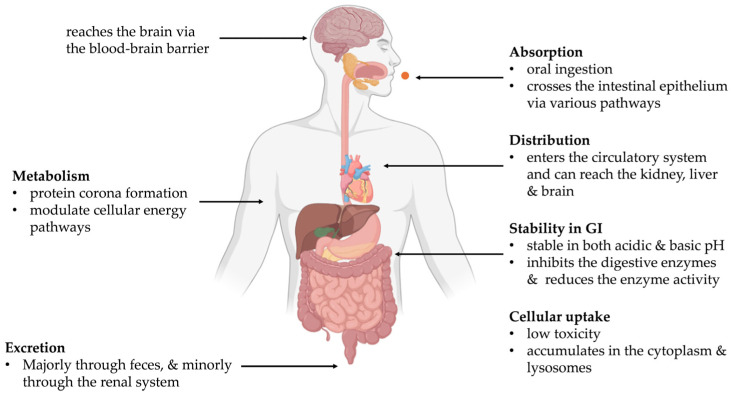
Biological fate of food-derived carbon dots (F-CDs).

Following oral ingestion, food-derived carbon dots (CDs) transit through the gastrointestinal (GI) tract, where their absorption is determined by physicochemical properties such as the particle size, surface charge (zeta potential), solubility, and surface functional groups [50], illustrated in Figure 2. Due to their ultrasmall size (<10 nm), CDs can cross the intestinal epithelium via passive diffusion, paracellular pathways, or active transport mechanisms such as clathrin-mediated endocytosis (CME) [50]. Research conducted using in vivo and in vitro models indicates that some of these nano-sized entities may enter the systemic circulation; for instance, fluorescent markers were detected in the blood within just 3 min post-administration [51]. Additionally, when CDs reach the bloodstream, they can target essential tissues, such as the liver, kidneys, and even the brain, which indicates their potential to cross highly selective physiological barriers [5].

### 4.2. Metabolism and Protein Corona Formation

Food-derived CDs are metabolically stable, with minimal degradation of their carbon core during GI transit [52]. However, they undergo surface modifications via oxidation and binding to biomolecules such as proteins and lipids. This leads to the formation of a protein corona, which alters nanoparticle recognition, cellular uptake, and biodistribution [53].

Metabolomic studies have shown that CDs can modulate cellular energy pathways. For instance, CDs from baked lamb impaired glycolysis and the tricarboxylic acid (TCA) cycle in PC12 cells, resulting in decreased ATP production and enhanced fatty acid biosynthesis [51]. Similarly, Maillard-reaction-derived CDs altered mitochondrial activity and shifted energy metabolism in HepG2 liver cells [52]. These findings indicate that, although CDs remain structurally intact, their surface reactivity can significantly affect host metabolic processes.

### 4.3. Excretion and Clearance

The majority of food-derived CDs are not absorbed and are excreted via feces, indicating low systemic bioavailability [54]. Only a small fraction (<5 nm particles) can enter circulation and undergo renal clearance, as confirmed by studies using fluorescent or radiolabeled CDs [55]. Their nanoscale size and hydrophilicity allow glomerular filtration, but this clearance pathway accounts for only a minor proportion of ingested CDs.

Animal studies (e.g., mouse and zebrafish models) report that most CDs are eliminated within 24–72 h after oral administration without long-term accumulation in tissues. Clearance kinetics are highly dependent on the particle size, surface charge, and functional groups (e.g., carboxyl or hydroxyl moieties) [56].

### 4.4. Stability in the Gastrointestinal Tract

The GI tract presents a challenging environment due to variable pH, digestive enzymes, and bile salts. Most food-derived CDs show stable fluorescence, size, and structure when exposed to simulated gastric and intestinal fluids [57]. Nonetheless, not all CDs are the same. Stability relies on precursor compositions and synthetic conditions, predominantly the temperature of pyrolysis. CDs synthesized from sugar-rich precursors or at lower pyrolysis temperatures may be less stable, exhibiting aggregation or partial enzymatic degradation [58].

Furthermore, the functionalities of CDs at the surface govern their interactions with the enzymes of digestion. As a case in point, pepsin can associate strongly with amine-functionalized CDs and inhibit enzymatic activity and alter digestion conditions [59]. Such interactions not only can govern local digestion but also can change the gut microbiota composition, with downstream biological effects, including inflammation or changes in permeability.

### 4.5. Cellular Uptake (In Vitro and In Vivo)

In vitro studies on intestinal (Caco-2), colon (HT-29), and liver (HepG2) cell lines have all indicated that CDs from foods are internalized via energy-dependent endocytic pathways, such as clathrin-mediated endocytosis. Upon internalization, CDs accumulate preferentially in the cytoplasm and lysosomes, with fluorescence tracking confirming their accumulation inside the cells [60]. Uptake is efficient without additional surface functionalization, which suggests intrinsic cell permeability.

Experiments in vivo, most of which are run on mouse and zebrafish models, give supplementary data. Upon oral or intravenous administration, CDs initially accumulate in the gut, liver, and kidneys, which gradually eliminate them through the kidneys and hepatobiliary excretion [61]. Protein corona formation and interactions with blood define the biodistribution profiles by modulating tissue uptake [53].

Importantly, acute toxicity is low at concentrations relevant to the diet, and cell viability remains exceedingly high for most test matrices [62]. Such findings bear testament to the potential safety of CDs during oral intake with thermally treated foods.

## 5. Interaction Between Food-Derived Carbon Dots and Gut Microorganisms

### 5.1. Composition of Gut Microbiota

The gut microbiota refers to the diverse community of microorganisms, including bacteria, archaea, fungi, viruses, and parasites, that inhabit the gastrointestinal tract. This intricate ecosystem plays a crucial role in maintaining host health by influencing metabolic processes, immune responses, and even behavior through the gut–brain axis. The balance of these microbial populations is essential for preventing diseases, as dysbiosis can lead to various gastrointestinal disorders and other health issues [63]. The gut microbiota comprises a variety of microorganisms, predominantly bacteria, but also includes archaea, fungi, and viruses [64]. This microbial community is highly diverse and varies significantly among individuals based on factors such as diet, lifestyle, antibiotics, genetics, and environment [63,65]. The most dominant species in the gut microbiota are primarily represented by members of the genus *Bacteroides*, particularly *Bacteroides thetaiotaomicron* and *Bacteroides vulgatus*. These species play crucial roles in the gut ecosystem, contributing to nutrient metabolism and host health [66]. The dominance of *Bacteroides* is influenced by their ability to degrade complex carbohydrates, such as glycosaminoglycans and mucins, which are abundant in the gut [67,68]. *Bacteroides thetaiotaomicron* is known for its role in polyamine biosynthesis; it is a key player in maintaining gut health through metabolic processes [68]. *Bacteroides vulgatus* is prevalent in various populations and contributes to the overall microbial diversity in the gut [69]. *Bacteroides* species assist in the fermentation of dietary fibers, producing short-chain fatty acids that are beneficial for gut health [67,70]. They also engage in mutualistic relationships with the host, enhancing nutrient absorption and immune function [71]. Conversely, while *Bacteroides* species dominate, other genera like *Bifidobacterium* also play significant roles in gut health, particularly in maintaining microbial diversity and robustness [72]. This highlights the complex interplay within the gut microbiota, where multiple species contribute to overall health. The gut microbiota contributes to metabolic functions, immune system modulation, and protection against pathogens [73,74]. Dysbiosis is linked to conditions like inflammatory bowel disease, colorectal cancer, and metabolic disorders [74,75]. Manipulating the gut microbiota through probiotics, prebiotics, and fecal microbiota transplantation shows promise in treating microbiota-related diseases [74]. While the gut microbiota is often viewed as beneficial, emerging research suggests that certain microbial populations can also contribute to disease under specific conditions, highlighting the complexity of host–microbiome interactions.

### 5.2. Role of Gut Microbiota in Health and Disease

The human gastrointestinal microbiota consists of trillions of microbes residing in the digestive tract. These microbes and their dysbiosis can maintain human health and contribute to the development of different diseases [76]. The gut microbiota plays a vital role in maintaining host health by contributing to a wide range of physiological functions. It supports key metabolic processes, including the fermentation of indigestible dietary fibers into short-chain fatty acids (SCFAs), synthesis of essential vitamins, and regulation of lipid and glucose metabolism [77]. In addition to its metabolic roles, the gut microbiota is central to immune system modulation, helping to shape both innate and adaptive immune responses [78]. It educates immune cells, promotes tolerance to beneficial microbes, and provides protection against pathogenic invasion by occupying ecological niches and producing antimicrobial compounds [74]. However, this balance can be disrupted. A condition known as dysbiosis—marked by reduced microbial diversity and a shift toward pathogenic bacteria—has been increasingly associated with the development of various diseases. These include inflammatory bowel disease (IBD), colorectal cancer, obesity, type 2 diabetes, and other metabolic disorders [74,75]. Dysbiosis contributes to disease through mechanisms such as increased intestinal permeability (leaky gut), chronic inflammation, and the generation of harmful metabolites like lipopolysaccharides (LPS), which can enter systemic circulation and disrupt physiological homeostasis [79].

Given the central role of the gut microbiota in both health and disease, therapeutic manipulation of the microbiome has become a promising avenue in modern medicine. Several recent studies focused on the close relationship between the gut microbiota and health or diseases from different point of views [80,81,82]. Strategies such as probiotics (live beneficial microbes), prebiotics (dietary components that selectively nourish beneficial bacteria), and fecal microbiota transplantation (FMT) are being actively explored and have shown success in managing microbiota-related conditions, particularly recurrent *Clostridioides difficile* infections, and are being investigated for use in IBD, metabolic syndromes, and even neurological disorders [74]. Despite the generally beneficial perception of the gut microbiota, emerging research emphasizes its context-dependent effects. Under certain conditions, even commensal bacteria can contribute to pathology. For example, microbial metabolites or an imbalanced immune response can transform a once harmless species into a pro-inflammatory agent [83]. This dual nature highlights the complexity of host–microbiome interactions, where health outcomes are not only determined by the presence or absence of specific microbes, but also by the broader ecological balance, host genetics, environmental factors, and microbial functions [84].

### 5.3. Impact of Carbon Dots on Gut Microbiota

The interaction between CDs and the gut microbiota is complex, with both detrimental and beneficial effects observed depending on the context of exposure. This interaction may be included in the dysbiosis induced by CDs and the protective effects of CDs, as presented in the following sub-sections.

#### 5.3.1. Dysbiosis Induced by Carbon Dots

Emerging research has demonstrated that CDs, depending on their source and structure, can induce significant dysbiosis in the gut microbiota, leading to a range of systemic health issues. One of the most concerning consequences is reproductive toxicity. The chronic ingestion of foodborne CDs has been shown to alter the gut microbial composition by decreasing the abundance of beneficial bacteria such as *Bacteroides acidifaciens* and increasing populations of harmful, pro-inflammatory microbes (Table 2). This microbial imbalance contributes to increased lipopolysaccharide (LPS) production [85], systemic inflammation [86], and damage to the intestinal barrier—factors that have been mechanistically linked to impaired reproductive function in male mice, including disrupted spermatogenesis and hormonal imbalance [87]. In addition to reproductive harm, CDs are also associated with metabolic disorders. Studies have revealed that chronic exposure to CDs can significantly impair glucose metabolism, leading to insulin resistance. These metabolic disturbances are mediated through CD-induced alterations in the gut microbiota, characterized by an increase in Gram-negative bacteria that produce inflammatory molecules like LPS and a concurrent decline in commensal species involved in maintaining gut barrier integrity and immune homeostasis. The resulting systemic inflammation interferes with insulin signaling pathways, exacerbating glucose intolerance and promoting the onset of metabolic syndrome [16].

Furthermore, certain types of engineered CDs can directly suppress the growth of probiotic strains, further contributing to gut dysbiosis. For example, carbon dots derived from ε-poly-L-lysine (PL-CDs) have been found to inhibit the proliferation of *Lactobacillus rhamnosus*, a key beneficial bacterium known for its role in maintaining mucosal immunity and intestinal homeostasis. This inhibition promotes a microbial imbalance and is associated with elevated markers of intestinal inflammation and barrier dysfunction [91]. The suppression of beneficial microbes not only weakens host defenses but may also create ecological niches that allow opportunistic pathogens to thrive. Collectively, these findings highlight the capacity of CDs to induce dysbiosis through multiple mechanisms—whether by promoting harmful bacteria, suppressing beneficial ones, or disrupting host–microbe interactions [92]. The downstream effects include reproductive toxicity, metabolic dysregulation, and chronic intestinal inflammation, emphasizing the need for comprehensive risk assessments of CDs, especially in long-term dietary exposures or medical applications. Understanding the microbial pathways affected by CDs will be essential in developing strategies to mitigate their adverse effects while harnessing their potential benefits [88].

#### 5.3.2. Protective Effects of Carbon Dots

Carbon dots, while often studied for their antimicrobial properties and potential toxicological effects, have also shown promising protective and therapeutic roles—particularly in the context of gut health and inflammation [89]. Emerging evidence suggests that specific types of CDs can exert anti-inflammatory effects and contribute to the restoration of the gut microbiota balance, indicating a dual nature that merits deeper exploration [88]. One key mechanism underlying these protective effects is the ability of certain CDs to modulate the composition of the gut microbiota [90]. For instance, citric acid and polyethylene polyamine-derived carbon dots (CP-CDs) have been shown to significantly alleviate intestinal inflammation in experimental models of colitis. This is achieved by increasing the relative abundance of beneficial bacterial genera, such as *Ligilactobacillus* and *Enterorhabdus*, while concurrently suppressing harmful taxa like *Clostridia_UCG_014* [89]. The resulting rebalancing of the gut ecosystem contributes to a healthier intestinal environment, enhancing mucosal integrity and reducing local immune activation.

In addition to microbial modulation, CDs have also demonstrated antioxidant properties that contribute to their therapeutic effects [90]). Carbon dot nanozymes—engineered CDs that mimic the activity of natural antioxidant enzymes—have been successfully developed to combat oxidative stress in models of colitis. These nanozymes can scavenge reactive oxygen species (ROS) in intestinal tissues, reducing oxidative damage and inflammation [88] In turn, this helps to preserve the structure and function of the gut barrier, preventing the translocation of microbial products like lipopolysaccharides (LPS), which are known to drive systemic inflammation [93]. These findings highlight the therapeutic potential of CDs not only as antimicrobial agents but also as modulators of the gut microbiota and inflammation [92]. Their unique physicochemical properties—such as small size, tunable surface chemistry, and biocompatibility—enable them to interact effectively with microbial communities and host tissues. Importantly, unlike broad-spectrum antibiotics, which indiscriminately disrupt microbial populations, CDs offer the possibility of more targeted interventions that preserve or even enhance the growth of beneficial bacteria [94]. However, the beneficial effects of CDs are not universal and appear to depend strongly on factors such as CD type, synthesis method, dose, and exposure duration. While some CDs can promote the microbial balance and reduce inflammation, others—particularly at higher doses or with certain surface modifications—can induce dysbiosis, damage the intestinal barrier, and trigger systemic immune responses [95]. This dual behavior underscores the complexity of CD–microbiota interactions and calls for a more nuanced understanding of their biological effects.

In conclusion, carbon dots represent a versatile class of nanomaterials with the potential to both harm and heal, depending on how they are designed and applied. Their demonstrated ability to restore the gut microbial balance, reduce oxidative stress, and attenuate inflammation opens the door to their use as therapeutic agents in gastrointestinal disorders such as inflammatory bowel disease (IBD). Nonetheless, their dual role necessitates careful optimization and rigorous evaluation to ensure safety and efficacy in clinical applications. Future research should aim to identify the specific structural and functional properties of CDs that confer protective versus harmful effects, ultimately guiding the development of targeted, microbiota-friendly nano-medicines.

### 5.4. Factors Affecting Carbon Dot Behavior Toward the Gut Microbiota

**Figure 3:**
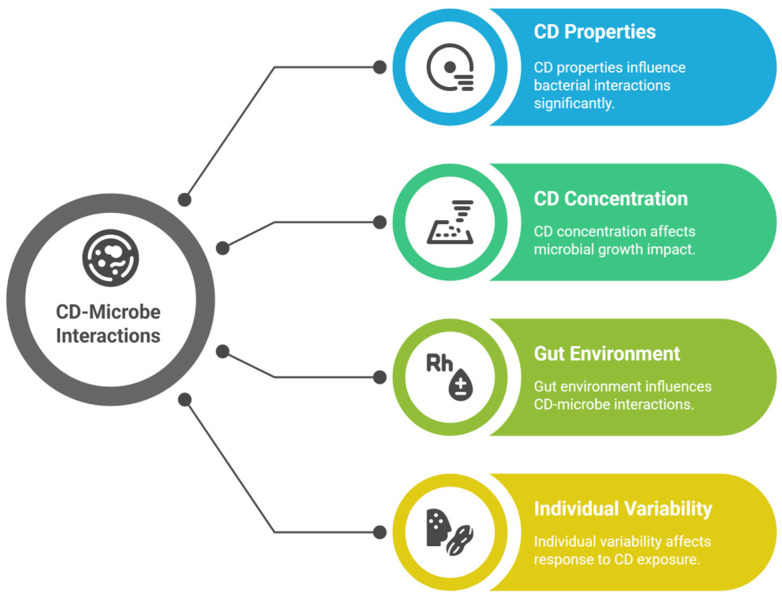
Main factors affecting the behavior and interaction of CDs toward the gut microbiota.

The behavior of CDs toward the gut microbiota is shaped by multiple interrelated factors, with their origin emerging as a foundational influence. CDs can be derived from a variety of sources, including food items commonly subjected to thermal processing. In one noteworthy study, CDs were isolated from commercial cola, representing a typical example of foodborne CDs formed during the high-temperature cooking or processing of carbohydrates (Table 3). This finding underscores the importance of the origin and synthetic route in defining the physicochemical properties of CDs—such as the particle size, surface charge, and functional groups—which ultimately govern their interactions with the gut microbiota [87]. Different food matrices and preparation conditions may produce CDs with unique structural and chemical characteristics, thereby altering their biological reactivity and potential toxicity. For instance, CDs formed from sugar-rich drinks may differ significantly from those derived from grilled meat or baked goods, affecting how they modulate microbial populations in the gut. Factors affecting CDs in the gut are summarized in Figure 3.

Equally important is the duration and frequency of exposure. Unlike acute exposure, which may have transient or minimal effects, chronic ingestion over an extended period appears to be critical in inducing lasting alterations in the gut microbiota. In the referenced study, a 15-week period of the continuous consumption of foodborne CDs led to profound changes in the gut microbial composition [89,97]. This suggests that the cumulative dose and persistence of CDs in the gut environment are crucial determinants of their biological impact. Prolonged exposure allows for gradual microbial adaptation, selective pressures, and shifts in the community structure, which may not be evident in short-term studies.

One of the key microbial consequences observed following chronic CD exposure is a shift toward a dysbiosis state. Specifically, there was a marked increase in the relative abundance of Gram-negative, lipopolysaccharide (LPS)-producing bacteria, such as members of the phylum Proteobacteria [73]. Concurrently, beneficial anti-inflammatory gut commensals like *Bacteroides acidifaciens* and *Akkermansia muciniphila* were significantly depleted [92]. These changes reflect a loss of microbial diversity and functional stability in the gut ecosystem, promoting the overproduction of LPS—a potent endotoxin associated with inflammation and metabolic dysfunction. The elevated levels of LPS not only compromise the integrity of the intestinal epithelial barrier but also facilitate its translocation into systemic circulation. This translocation is associated with low-grade systemic inflammation, a condition often implicated in metabolic disorders and reproductive dysfunction. Inflammatory responses triggered by microbial dysbiosis can disrupt hormonal signaling, impair spermatogenesis, and damage reproductive organs, suggesting a plausible link between the gut microbial imbalance induced by CDs and the development of reproductive toxicity [87].

Thus, the gut microbiota serves as a central mediator of the biological effects of CDs. Its composition and resilience determine whether CDs are tolerated, neutralized, or capable of triggering a cascade of adverse effects. These findings highlight the complex interplay between nanomaterials and host-associated microbial communities, emphasizing the need for comprehensive risk assessments, especially in populations with pre-existing metabolic vulnerabilities. Further research is warranted to explore the threshold levels, structural determinants, and possible mitigation strategies—such as the use of probiotics or dietary interventions—to counteract the gut-related toxic effects of chronic CD exposure.

### 5.5. Structural Properties and Surface Functionalization of CDs, Controlling Their Interaction with the Gut Microbiota

The structural characteristics of CDs play a pivotal role in determining their interactions with microorganisms, positioning them as highly promising candidates for antimicrobial applications. These interactions are governed by a combination of physical and chemical properties, including size, morphology, surface chemistry, amphiphilicity, surface charge, and their capacity to generate reactive oxygen species (ROS). One of the defining features of CDs is their extremely small size, typically less than 10 nm [98,99,100]. This nanometric scale enables them to penetrate microbial cell walls with ease, allowing for close interactions with intracellular structures and contributing directly to cellular disruption and death [101]. Their morphology also plays a role, as uniform and spherical particles tend to exhibit more predictable diffusion and cellular uptake. Surface functionalization adds another layer of complexity and adaptability. By chemically modifying the surface of CDs, their biocompatibility can be enhanced, and their interaction with specific microbial targets can be improved. Functional groups, especially those containing oxygen or nitrogen, increase hydrophilicity and microbial adhesion, allowing for more effective contact with bacterial membranes [102,103,104]. Moreover, attaching antimicrobial agents such as sulfanilamide to CDs has been shown to increase selectivity, enabling targeted action against specific microbial strains, such as Gram-positive bacteria [105].

Amphiphilicity is another crucial feature of CDs that enables them to disperse well in biological environments by interacting with both hydrophilic and hydrophobic domains. This property enhances their distribution in physiological fluids and tissues, which is critical for effective antimicrobial performance [106]. In addition, the surface charge of CDs influences their interaction with microbial membranes. Positively charged CDs are more likely to bind to negatively charged bacterial cell walls, disrupting membrane integrity and causing the leakage of intracellular contents [106]. Perhaps one of the most potent antimicrobial mechanisms of CDs is their ability to generate reactive oxygen species upon exposure to light. These photogenerated ROS—including hydroxyl radicals, singlet oxygen, and superoxide anions—can oxidize essential microbial biomolecules such as lipids, proteins, and DNA, ultimately leading to cell death [101,106]. This light-triggered antimicrobial effect makes CDs particularly attractive for use in photodynamic therapy and sterilization technologies. Despite their promising antimicrobial properties, several challenges remain. One of the main concerns is the potential toxicity of CDs to human cells, which necessitates careful control over the dosage, surface chemistry, and exposure conditions. Moreover, while laboratory studies have shown encouraging results, further research is needed to fully understand their long-term safety, biodegradability, and behavior in complex biological systems.

### 5.6. Mechanisms of Antimicrobial Action of Carbon Dots

**Figure 4:**
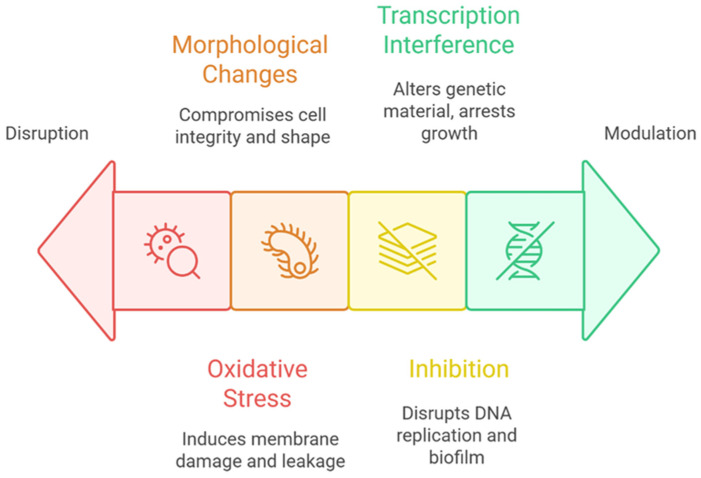
Potential mechanisms of carbon dot (CD) interactions with gut microbiota.

The antimicrobial action of CDs is multifaceted, involving a range of physical and biochemical mechanisms that collectively disrupt microbial viability and function [88]. At the core of this activity is the ability of CDs to generate reactive oxygen species (ROS), especially under light exposure. These ROS—including hydroxyl radicals and singlet oxygen—induce oxidative stress within bacterial cells, leading to lipid peroxidation of the cell membrane, structural rupture, and the leakage of cytoplasmic contents [107,108]. This oxidative damage not only compromises membrane integrity but also interferes with vital cellular homeostasis. In addition to ROS-mediated damage, CDs exert direct physical effects on bacterial cells. Their interaction with cell membranes causes significant morphological alterations, such as elongation, membrane roughening, and irregular surface protrusions. These changes reflect mechanical stress and membrane destabilization, further contributing to the breakdown of the cellular structure and eventual cell death [107]. CDs also disrupt essential intracellular processes. By penetrating bacterial cells, they can inhibit DNA replication and interfere with other critical metabolic functions. Notably, studies have shown that CDs can impair quorum sensing (QS)—the bacterial communication system that regulates gene expression in response to population density. For example, in *Staphylococcus aureus*, CDs suppress QS-regulated genes, leading to impaired biofilm formation and communication-dependent growth [109]. Furthermore, CDs can modulate bacterial gene expression more broadly, altering transcriptional activity and disturbing core metabolic pathways in organisms such as *Escherichia coli*, ultimately resulting in growth arrest and apoptosis (Figure 4; [109,110]).

Beyond their direct antimicrobial effects, CDs also impact the broader microbial ecosystem, particularly in the gut. Chronic exposure to CDs has been shown to induce gut microbiota dysbiosis, marked by an increase in harmful Gram-negative bacteria and a decline in beneficial species like *Bacteroides acidifaciens* and *Akkermansia muciniphila* [111]. This microbial imbalance leads to the overproduction of lipopolysaccharides (LPS), which compromise the intestinal epithelial barrier, facilitate the translocation of endotoxins into systemic circulation, and trigger systemic inflammation. The disruption of QS mediated by CDs is a key factor in these gut-related outcomes. Quorum sensing plays a vital role in maintaining the structure, function, and stability of the gut microbiome. It governs inter-bacterial communication and regulates processes essential for colonization, nutrient metabolism, immune modulation, and resistance to pathogens [112,113]. When CDs interfere with QS signaling, they impair bacterial coordination and gene regulation, potentially leading to compromised gut barrier function, inflammation, and susceptibility to conditions such as inflammatory bowel disease, metabolic syndrome, and immune dysregulation [114,115].

While this disruption can lead to negative health consequences, it also reveals a therapeutic opportunity. The ability of CDs to modulate QS suggests potential for their use in controlling pathogenic bacteria, particularly in infections where biofilm formation and virulence are QS-dependent. By selectively targeting QS pathways, it may be possible to inhibit harmful microbial behaviors while preserving beneficial microbes, offering a novel strategy for managing microbial imbalances and restoring gut health [113,116]. Despite their promise, the application of CDs as antimicrobial agents must be approached cautiously. Their potential cytotoxicity to human cells, dose-dependency, and long-term biological effects remain areas of concern. Future research should focus on optimizing the physicochemical properties of CDs—such as the size, surface charge, and functionalization—to enhance selectivity, reduce host toxicity, and maximize therapeutic benefits.

## 6. Knowledge Gaps and Future Research Directions

Despite the growing interest in food-derived carbon dots (CDs) and their interactions with the gut microbiota, several critical knowledge gaps remain that limit their responsible application and risk evaluation. One of the foremost challenges is the lack of specificity and standardization in analytical detection. Many current methods do not reliably distinguish CDs from other fluorescent or carbonaceous species formed during food processing, especially in complex matrices like cooked meats, sauces, or fermented products. Standardized protocols, reference materials, and validated analytical pipelines are urgently needed to confirm CD identity and quantify exposure accurately.

Another major limitation is the insufficient understanding of long-term biological fate. While acute exposure studies show limited toxicity, the effects of chronic, low-dose ingestion—especially in vulnerable populations—are poorly characterized. There is a lack of in vivo models simulating realistic dietary intake patterns over months or years. Additionally, tracking the biodistribution and biotransformation of food-derived CDs in humans remains a technical challenge due to the absence of reliable labeling techniques and imaging markers.

The role of CDs in modulating the gut microbiota and host–microbiome interactions is still emerging and primarily based on animal studies. Human-relevant data, including from microbiota-depleted or personalized gut models, are largely unavailable. Moreover, existing studies often fail to control for diet, the microbial baseline, and other confounding variables that influence gut microbial dynamics. This restricts the ability to generalize findings or determine population-specific risks and benefits.

On the regulatory side, no specific frameworks currently address the safety or permissible levels of foodborne CDs. Most food safety agencies treat these nanoparticles under broader nanomaterial regulations, which do not account for their unique formation pathways, structure–function relationships, or microbiota-mediated effects. As a result, risk assessments remain incomplete, and labeling guidelines for consumers are absent. Currently, no maximum allowable intake (MAI) levels for food-derived CDs have been established by regulatory authorities. Defining such thresholds will require standardized toxicological studies, realistic exposure assessments, and harmonized international guidelines to ensure consumer safety.

Future research should focus on developing interdisciplinary approaches that integrate nanotechnology, microbiology, toxicology, and food science. Key priorities include the following: (i) longitudinal in vivo studies with microbiota profiling, (ii) structure–activity relationship mapping of CDs, (iii) improved detection and tracking technologies, (iv) harmonized toxicity testing protocols, and (v) the design of intelligent delivery systems to minimize non-specific accumulation in vivo. Promising strategies include surface functionalization with targeting ligands (e.g., antibodies, peptides, or aptamers), stimuli-responsive systems triggered by pH, enzymes, or redox conditions, and biomimetic coatings such as cell membrane cloaking, which have been successfully applied in nanomedicine but remain underexplored for food-derived CDs. In parallel, computational modeling and AI tools could be employed to predict CD–microbiome interactions based on physicochemical properties.

Addressing these knowledge gaps will be essential to ensure the safe development, regulation, and potential therapeutic use of food-derived carbon dots, while also mitigating unforeseen health risks.

## 7. Conclusions

Food-derived carbon dots (F-CDs) represent an emerging class of nanostructures that are unintentionally generated during common food processing steps such as heating, roasting, fermentation, and acid-mediated reactions. Their unique optical and chemical properties, combined with their widespread occurrence in daily diets, have raised increasing interest regarding their potential roles in food safety and human health. Current evidence suggests that F-CDs can interact with the gut microbiota and host metabolism, but the available data are still fragmented and primarily based on in vitro or animal studies. Moreover, their long-term biological fate, toxicity under chronic exposure, and regulatory status remain poorly defined.

To ensure safe and beneficial applications, research must now focus on several overarching priorities. First, standardized detection methods and validated reference materials are urgently needed to differentiate F-CDs from other carbonaceous species in complex food matrices. Second, longitudinal in vivo studies are required to assess their biodistribution, metabolism, and potential cumulative effects. Third, mechanistic investigations should clarify how F-CDs modulate gut microbial diversity, metabolite production, and host signaling pathways. Finally, future research should integrate nanotechnology and food science to design intelligent delivery and control strategies, thereby reducing non-specific accumulation in vivo and enabling safe applications.

F-CDs are not only a novel analytical and toxicological challenge but also a potential opportunity for advancing food nanoscience. With interdisciplinary collaboration, their risks can be better managed and their unique properties potentially harnessed for beneficial applications in food safety, nutrition, and health.

## Figures and Tables

**Table 2:** Food-derived carbon dots (F-CDs) and gut microbiomes.

CD Source	Microbial Species Affected	Effects	Proposed Mechanism	Refs.
Histidine-derived carbon dots (His-CDs)	Increase the abundance of beneficial bacteria in the intestinal tract (*Firmicutes* and *Bacteroidetes*)	Anti-inflammatory bowel disease (IBD)	CDs have antioxidant activity for scavenging free radicals, thereby exhibiting a relieving effect on IBD-caused oxidative stress	[88]
Metal-free carbon dots (CP-CDs)	Increase the abundance of Firmicutes, and a decrease in *Proteobacteria* and *Fusobacteriota*	Anti-inflammatory bowel disease (IBD)	CP-CDs regulate oxidative stress levels and effectively down-regulate inflammatory cytokines in the colon	[89]
N-doped carbon dot nanozyme (CDzyme)	Modulate gut microbials (*Eubacterium coprostanoligen*)	Alleviate stress-induced depression	Restores the gut microbiota composition, develop intestinal flora dysbiosis nano-medicine for oxidative-stress-related multifactorial diseases	[90]
Foodborne carbon dots (CDs)	Increases the abundance of Gram-negative bacteria (*Proteobacteria* and *Desulfovibrionaceae*)	Induces glucose homeostasis imbalance	CDs increase fasting glucose levels and exacerbate liver and intestinal barrier damage	[16]
Foodborne carbon dots (CDs)	Increases the abundance of harmful bacteria (*Proteobacteria*, *Oscillospira*, *Desulfovibrionaceae*, and *Ruminococcaceae*)	Induces inflammation-mediated insulin resistance	Increases pro-inflammatory bacteria, activates systemic inflammation, and induces hepatic insulin resistance	[16]
CDs-poly-L-lysine (PL-CDs)	Regulates negatively growth of *Lactobacillus rhamnosus*	Induces inflammatory infiltration	PL-CDs induce pathological damage to the intestine causing intestinal flora dysbiosis and intestinal inflammation	[91]

**Table 3:** Applied foodborne CDs under different conditions and their impact on microbiota and health.

CD Type	Study Type (In Vitro/In Vivo)	Health Impact	Observed Microbiota Change	Refs.
CDs investigated in this study were isolated from commercial cola	In vivo study, using male mice to assess the effects of CDs and involving a 15-week investigation	Chronic CD exposure: Triggers gut dysbiosis → systemic inflammation → metabolic and reproductive disorders, especially reduced male fertility	Harmful bacteria: Increased LPS-producing bacteria, promoting gut barrier damage and inflammation Beneficial probiotics: Reduced levels of *B. acidifaciens* and *A. muciniphila*, key anti-inflammatory species	[87]
Foodborne CDs from self-brewing beer	In vivo mouse model	Elevated LPS crossed a weakened gut barrier, activating liver inflammation (TLR4/NF-κB/MAPK) and worsening insulin resistance, liver, and gut damage. Effects were blocked by antibiotic	CDs increased Gram-negative bacteria (*Proteobacteria*, *Desulfovibrionaceae*) and LPS-synthesis pathways (e.g., lipid A biosynthesis)	[93]
Foodborne carbon dots	In vivo (chronic CD exposure in mice, including FMT and microbiota-depleted models)	Gut dysbiosis led to elevated LPS, intestinal and liver inflammation via TLR4/NFκB/MAPK pathway, and insulin resistance; effects reversed by probiotics and absent in microbiota-depleted mice	Reduced *Bacteroides*, *Coprococcus*, S24-7; increased *Proteobacteria*, *Oscillospira*, *Desulfovibrionaceae*, *Ruminococcaceae*; higher Firmicutes/Bacteroidetes ratio	[92]
Carbon quantum dots (CQDs)	In vivo (chronic exposure in common carp for 5 weeks)	CQDs accumulated in liver and intestine, causing tissue damage, reduced antioxidant enzymes, increased MDA, downregulated nrf2/ho-1, activated TLR pathways, and disrupted lipid metabolism	Decreased gut microbiota diversity and richness; increased harmful and decreased beneficial bacteria; microbial shifts correlated with antioxidant capacity, immune response, and lipid metabolism	[73]
Epsilon-poly-L-lysine-based CQDs (PL-CDs), prepared via pyrolysis	In vitro (probiotic modulation) and in vivo (chronic exposure in common carp over 5 weeks)	CQDs accumulated in liver and intestine, causing tissue damage, oxidative stress (↓ SOD, GSH-PX; ↑ MDA), downregulated nrf2/ho-1, activated TLR pathways, disrupted lipid metabolism, and impaired intestinal barrier	Reduced diversity and richness; decreased beneficial and increased harmful bacteria; higher Firmicutes/*Bacteroidota* ratio; increased *Lachnospiraceae*, decreased *Muribaculaceae*; microbial shifts linked to antioxidant, immune, and lipid functions	[91]
Metal-free CP-CDs (citric acid + polyethylene polyamine)	In vitro (cells, nematodes) and in vivo (DSS-induced colitis in mice)	Rebalanced gut microbiota, reduced oxidative stress and inflammatory cytokines, restored intestinal barrier, lowered DAI score, and alleviated colitis symptoms in mice	Increased beneficial bacteria (*Ligilactobacillus*, *Enterorhabdus*); decreased harmful bacteria (unclassified_Clostridia_UCG_014)	[89]
Bacterially-derived CQDs, attached to *Bifidobacterium infantis* via silica nanoparticles	In vitro (intestinal epithelial model colonized by *E. coli*)	Enhanced *Bifidobacterium infantis* survival and adhesion; improved probiotic functionality	No direct observation of overall microbiota shifts; focused on *Bifidobacterium infantis* interaction with *E. coli*-colonized epithelial layers. increased *E. coli* killing via ROS generation	[96]

## Data Availability

No new data were created or analyzed in this study. Data sharing is not applicable to this article.
